# Assessment of Perinatal Depression Risk among internally displaced Yazidi Women in Iraq: a descriptive cross-sectional study

**DOI:** 10.1186/s12884-022-04658-3

**Published:** 2022-04-25

**Authors:** Pegah AM Seidi, Nazdar Qudrat Abas, Dilshad Jaff, Raven Dunstan, Lein Soltan, Amanda Brumwell, Michael Wilson, Thomas Nicholson, Aunchalee E. L. Palmquist

**Affiliations:** 1 Research Center, University of Garmian, Kurdistan, Iraq; 2 Research Center, University of Garmian Kalar, Kurdistand, Sulaimania Iraq; 3 Gillings School of Global Public Health, University of North Carolina at Chapel Hill, Chapel Hill, NC USA; 4 Advance Access & Delivery, Durham, NC USA

**Keywords:** Yazidi, humanitarian, genocide, violence, postpartum depression, perinatal mood disorder, internally displaced persons, perinatal, EPDS, Iraq

## Abstract

**Background:**

Yazidi survivors of a 2014 genocidal attack by the self-proclaimed Islamic State of Iraq and Syria (ISIS) have complex medical and mental health needs in the perinatal and postpartum period. Few studies have assessed perinatal mental health needs for this population of women who are living in camps for Internally Displaced Persons (IDP) in the Kurdistan Region of Iraq (KRI).

**Methods:**

The specific aim of this formative cross-sectional study was to assess the prevalence of perinatal depressive symptoms, specifically the risk of perinatal depression symptoms, among a purposive sample of Yazidi women living in camps for internally displaced persons in the Kurdistan region of Iraq. One hundred twenty-two pregnant and recently postpartum (<1 year) Yazidi women completed a Kurdish-language version of the Edinburgh Postnatal Depression Scale (EPDS) questionnaire. Pregnant and postpartum participants’ responses were analyzed together, in order to assess an overall combined risk of perinatal mental health issues for the study population. Logistic regression analyses were used to measure the association of participant characteristics with an elevated risk of perinatal depressive symptoms.

**Results:**

Participants were 17-45 years of age (mean 32 years, SD 7.63) Among the 122 women, 67.2% (n=82) were pregnant and 32.8% (n=40) were <1 year postpartum. Overall, 78% (n=95) of participants were at an elevated risk of depression (EPDS >10), and 53% (n=65) of all participants were at risk of moderate to severe depression (EPDS >12). Thoughts of self-harm (EPDS item 10) were reported among 97% (n=118) of participants. Logistic regression analysis indicated that increased risk of perinatal depressive symptoms was significantly associated with reports of health problems during pregnancy (OR=3.22, 95% [CI]:1.08-9.61) and marital status (OR=16.00; 95% [CI]: 0.42-0.50). Age (OR= 0.84; 95% [CI]: 0.75–0.94) and level of education (OR=0.15; 95% [CI]: 0.42-0.50) had protective effects.

**Conclusions:**

Rates of perinatal depressive symptoms risk among internally displaced Yazid pregnant and postpartum women are higher than the general Kurdish-speaking population in Iraq (28.4%). Culturally responsive trauma informed perinatal and postpartum care services, which include both community-based and clinical strategies for perinatal depressive symptoms and suicide prevention for this population, are critically needed.

## Background

The World Health Organization (WHO) recognizes maternal mental health as an important determinant of maternal and child morbidity and mortality [1]. Supporting mental health throughout pregnancy and the postpartum period is considered a global public health priority, and untreated perinatal mental health issues are related to poorer health outcomes for both mothers and their infants [2, 3]. Mental health disorders in pregnant and postpartum women commonly include stress, anxiety, depression, and psychosis, inter alia [4]. perinatal depressive symptoms, a non-psychotic depressive and major disabling mood disorder, is one of the most common complications featured in the Diagnostic and Statistical Manual of Mental Disorders (DSM) for women during their childbearing years [5]. Edinburgh Postnatal Depression Scale (EPDS) has been used very widely to measure depression for women during the perinatal period [6, 7]; the perinatal period is defined as the time period from one year before to 18 to 24 months after a child’s birth [8]. Perinatal mood disorders include depression onset during pregnancy and after giving birth [9]. Untreated, perinatal depression may have immediate and long-term and intergenerational health effects, for example negative effects on breastfeeding outcomes and the newborn’s nutrition [10], cognitive, language and physical developmental delay, and future mental health [3].

The global average rate of reported perinatal depressive symptoms ranges from 1.9 to 82% in high-income countries (HIC) and from 5.2 to 74% in low- and middle-income countries [11]. Rates of postnatal depression for women migrants [12], asylees [13], and refugees are considerably higher when compared to the general population [14]. Internally displaced persons (IDPs), which are populations who are forced to seek refuge within the political borders of their own country, often due to armed conflict, generalized violence, human rights violations, or natural disasters. IDPs are prioritized for humanitarian protection and assistance due to conditions of displacement that deprive people of basic needs for survival [15]. However, they are often displaced to areas where humanitarian assistance is difficult to implement, leaving many basic needs unmet. Among the approximately that around 55 million people worldwide who are internally displaced nearly half are women and children [16]. Gender is a critical dimension of health inequities among IDPs. IDPs who are women encounter more deprivation, insecurity, and physical, sexual, and psychological violence compared to IDPs who are men [17].

Nearly 1.2 million people are internally displaced in Iraq, with more than four million IDP returnees documented in 2021 [18]. Yazidis comprise one of the largest IDP populations in Iraq. They are a Kurdish speaking religious and ethnic minority population in Iraq who has experienced centuries-long persecution, enslavement, violence, and discrimination due to various extremist Islamization campaigns throughout the Middle East. It is estimated that over the last 800 years, approximately 1.2 million Yazidis residing in Iran, Iraq, Syria, and Turkey have been killed during genocidal attacks on the Yazidi people, their religion, culture, and livelihoods [19–21].

In August 2007, two Yazidi villages near Mosul were completely destroyed in an explosion by Al-Qaeda, it left 796 deaths and 1,562 injures [22]. Beginning in 2014, targeted violence against Yazidis in Iraq escalated during systematic attacks in Sinjar, Zumma, and the Ninewa plains that were led by the self-proclaimed “Islamic State of Iraq and Syria (ISIS),” or Daesh [23]. On August 3rd, ISIS began separating men and boys over the age of 12 and forcing them to convert or be executed. Women and other children were forced to witness these killings before they were also separated and forcibly transferred to Iraq and Syria. ISIS abducted at least 5,800 Yazidi women and girls and sold them into sexual slavery [24]. By March 2015, 500,000 Yazidis, predominantly from the Sinjar region, had been displaced [25]. A 2016 report issued by the international Commission of Inquiry on the Syrian Arab Republic describes the atrocities committed by ISIS against the Yazidi people, recognizing it as genocide on the basis of “killings; sexual slavery; enslavement, torture and inhuman and degrading treatment and forcible transfer causing serious bodily and mental harm; the infliction of conditions of life that bring about slow death; the imposition of measures to prevent Yazidi children from being born, including forced conversion of adults, the separation of Yazidi men and women, and mental trauma; and the transfer of Yazidi children from their own families and placing them with ISIS fighters, thereby cutting them off from beliefs and practices of their own religious community [26].”

The long-term and intergenerational effects of these events are acute [27]. Survivors and members of the Yazidi community, including children, emigrees, and women and girls, commonly report persistent severe psychiatric sequalae [20, 28]. A recent study reported that “more than 80% of Yazidi girls and women, and almost all participants who were formerly enslaved, met criteria for a probable DSM-5 PTSD diagnosis [29].” Other research showed similar results for all Yazidis, but especially women and girls [20, 30–32]. Although reports estimate that the average rate of perinatal depressive symptoms for Iraqi women in the general population is between 28.4% and 31.5%, there is a paucity of information regarding perinatal depressive symptoms and maternal mental health of Yazidi IDPs [33, 34]. Recognizing that personal history of mental illness is one of the most relevant factors associated with antenatal depression or anxiety, the maternal mental health of Yazidi women needs urgent attention [35].Therefore, this study aimed to measure the prevalence of perinatal depressive symptoms risk among Yazidi women living in IDP camps in the Kurdistan Region of Iraq (KR-I) and to identify factors associated with increased risk of perinatal depressive symptoms.

## Methods

### The aim, design and setting of the study

The current study was part of a larger formative mixed-methods investigation to understand the experiences and contextual factors affecting perinatal health and well-being, including gaps between documented perinatal health care needs and services available, to Yazidis living in IDP camps in remote areas of the KR-I. This cross-sectional quantitative study was carried out in the Ashti camp located near Sulaymaniyah Province of the KR-I. Ashti camp is one of the most unique IDP camps in KR-I and in the world because of the unique diversity of communities living alongside one another, including Sunni Arabs, Yazidis, Shabaks, and Turkmen. Ashti camp is the temporary home of 2,800 internally displaced families, comprised of more than 14,000 individuals. Within Ashti camp at the time of this study, there were 200 Yazidi families. Data were collected in person between September 30, 2019 and March 3, 2020 by P.S. and N.Q. and with the support of an on site social worker, who had developed rapport with the participants and was in a position to facilitate both data collection and referrals to available mental health supports as needed.

### Study participants and process

In total, 140 Yazidi women were invited to participate in the study according to inclusion criteria (being pregnant, or having a baby less than one year old, aged between 18 and 48 years, able to speak or read Kurdish). Those with any complication during or after pregnancy, mothers with a newborn requiring specialized neonatal medical care, and those whose baby had died were excluded from the study. The participants were not at captivity with IS. Data collection took place in two camps’ management offices in Ashti Camp through a personal interview with investigators.

#### Ethics approval

 was obtained from the Institutional Review Board of the University of North Carolina at Chapel Hill (IRB 18-0640) as well as local approval from the central office of camps managements in Sulaymaniyah Province and from the University of Garmian. All methods were performed in accordance with the relevant guidelines and regulations.

 After introducing the investigators, the ethical considerations of the study were explained verbally by the social worker in the preferred language of the participants. Consistent with the stipulations of the IRB-approved research protocol, all participants provided verbal consent to participate. Of 140 selected women,122 women consented to the study and participated during the study period.

### Measures

Data were collected using two structured surveys: [1] a socio-demographic survey, covering 5 items related to women’s age, pregnancy situation (pregnant, not pregnant but having a child less than 12 months); marital status (spouse alive/ dead/ divorced); number of children; and their total number of pregnancies; and [2] the Kurdish language version of the Edinburgh Postnatal Depression Scale (EPDS) which has been used previously with Kurdish-speaking populations in Iraq, specifically. Our team of humanitarian mental health experts - comprised of a Ph.D. psychology researcher, a psychiatric nurse, a frontline humanitarian physician, and a medical anthropologist with expertise in conducting cross-cultural perinatal health research - reviewed the Kurdish-language instrument and associated publications of its use in Iraq, piloted the EPDS along with semi-structured interviews with 30 internally displaced Yazidi IDP women, and made a final determination that it was a culturally responsive self-report measure appropriate for use in screening Yazidi IDP women at high risk of perinatal depressive symptoms. The women were interviewed face to face and the questions were asked and answers were reported directly by the interviewers. Indeed, the EPDS has been translated into numerous languages and used effectively for perinatal depressive symptoms screening at the primary health care level [36, 37]. Although the EDPS has an anxiety subscale, the validity of the subscale for this population was less clear, and so the research study focused specifically on risk of perinatal depressive symptoms. All participants in the study were connected with the on-site social worker and received information on referrals to any available mental health support services for Yazidi IDPs.

The EPDS consists of 10 items, and each item has four possible answers that are scored on a four-point scale (from 0 to 3). Prior research indicates that scores of 13 or higher convey a high sensitivity, specificity, and positive predictive power for perinatal depressive symptoms [37, 38]. The instrument has been translated into both Arabic and Kurdish evaluated for its appropriateness in Iraq [33]. The Cronbach’s coefficient was 0.73, which is acceptable. Although Edinburgh Postnatal Depression Scale (EPDS) was originally designed as a self-administered tool, previous research demonstrated that the interview of EPDS is equally valid for screening perinatal depression [39].

### Statistical analysis

Data analysis carried out using Statistical Package for the Social Sciences-22 (SPSS-22). Data, including 122 cases, obtained from the field were checked and input into SPSS-22 by a blind statistician. Frequency, means, and standard deviations were analyzed for the demographic variables and internal consistency for EPDS was assessed using Cronbach’s α. Univariate and bivariate analysis were conducted for all socio-demographic variables to explore any associations with perinatal depressive symptoms. Those variables that had a statistically significant relationship with perinatal depressive symptoms were included in a multivariate analysis using logistic regression to determine the factors associated with perinatal depressive symptoms and to adjust for confounders (P < 0.05).

## Results

### Demographic characteristics

Of 140 Yazidi women eligible for participation, 122 women consented and participated in the study (response rate: 87.14%). The socio-demographic characteristics of the sample are displayed in Table 1. The participants ranged from 18 to 45 years old, with a mean age of 31.68 (SD=7.63), 12.3% were widows,16.4% experienced miscarriage or abortion. and 67.2% reported health-related problem(s) during pregnancy. Only fifteen (12.3%) of the participants reported a higher education level. The majority of participants had four or more children (45.9%) and were pregnant four or more times in their lifetime (51.6%).

**Table 1:** Demographic characteristics of 122 Yazidi women study participants in Ashti IDP Camp

	Frequency	Percent
**Marital status**		
Spouse Alive	107	87.7
Widow	15	12.3
**Education**		
Basic	53	43.4
High school	54	44.3
Higher education	15	12.3
**Number of Pregnancies**		
First	19	15.6
Second	18	14.8
Third	22	18.0
Fourth or more	63	51.6
**Parity**		
One child	23	18.9
Two children	21	17.2
Three children	22	18.0
Four or more children	56	45.9
**Health problem(s) during pregnancy**		
Yes	82	67.2
No	40	32.8
**Miscarriage/Abortion**		
Yes	102	83.6
No	20	16.4
**Age -**	Mean	SD
	31.68	(7.63)

### Prevalence and risk factors of perinatal depressive symptoms

According to the results, 65 Yazidi women had a score of 13 or higher on the EPDS, which indicates the probable depression of 53.3% women, and 57 Yazidi women (46.7%) showed scores less than the cutoff point of 12. Of the 30 Yazidi women (24.6%) scoring between 10 and 12, which refers to possible depression. The other 27 Yazidi women (22.1%) showed no probability of depression. Results of suicidal thoughts (EPDS, question 10) showed that 61.5% of the women reported the thought of harming themselves ‘‘quiet often’’ and 28.6% responded ‘‘sometimes’’. Only 3 participants (2.5%) reported no thought of harming themselves. Among the 82 pregnant women, 60 women (73.2%) and among the 40 postpartum women 35 women (87.5%) had a score of EPDS >12.

The results of bivariate analysis showed that postnatal depression was significantly associated with the following socio-demographic factors: younger age, lower level of education, being a widow, and having fewer children and the following health-focused issues: lower parity, health-related problem(s) during pregnancy, and miscarriage or abortion. These characteristics age associated with higher risk of depression. According to logistic regression analysis, postnatal depression was statistically significantly associated with health problem(s) during pregnancy (OR=3.22; 95% confidence interval [CI]:1.08-9.61) and marital status (OR=16.00; 95% confidence interval [CI]: 0.42-0.50) (Table 2). Age (OR= 0.84; 95% confidence interval [CI]: 0.75–0.94) and level of educational (OR=0.15; 95% confidence interval [CI]: 0.42-0.50) had protective effects (Table 2).

**Table 2:** Logistic regression analysis of risk factors for perinatal depressive symptoms for study participants in Ashti IDP Camp

	B	S.E.	P	OR	95% CI
Age	-0.20	0.05	0.000	0.81	0.73-0.91
Level of education	-2.18	0.58	0.000	0.11	0.03-0.35
Marital status	2.77	1.34	0.040	16.00	1.14-224.28
Number of children	-0.19	0.37	0.609	0.82	0.39-1.72
Number of pregnancies	-0.10	0.37	0.777	0.90	0.43-1.87
Miscarriage or abortion	1.42	1.25	0.255	4.16	0.35-48.70
Other health-related problem(s)	1.17	0.55	0.035	3.22	1.08-9.61

## Discussion

Yazidi women survivors who have experienced pregnancy and birth while internally displaced have serious complex medical, mental health, and social support needs. Our study found a high prevalence of perinatal depressive symptoms risk, probable depression, and elevated risk of suicide as measured by the EPDS, which is consistent with other studies in the literature. Within the broader literature, estimates of the prevalence of general depression amongst displaced populations vary, which may be attributable to the use of varying analytical tools to measure depression [38, 40, 41]. Research in populations exposed to genocide have drawn attention to the gendered nature of mental health burdens, with women experiencing a more than two times higher prevalence of depression compared to men [42].

Although the Yazidi women who participated in the study were not asked to provide information regarding their personal experiences or exposures to genocidal violence or intergenerational trauma, it is clear from the broader literature that most Yazidis living in IDP camps have been affected by these atrocities both directly and indirectly. It is also well-established that the conditions of life in IDP camps often perpetuate increased exposures to gender-based violence and mental health issues. Other studies have found that Yazidi women and girls formerly enslaved by ISIS experienced a high prevalence of depression (88.1%) and that there was an association between the number of potential traumatic events (PTEs) experienced and depression [43]. Another study found that there were more displaced Yazidi women with major depressive disorder than displaced Yazidi men, which was associated with a higher self-reported exposure to war-related events [17]. Furthermore, the literature emphasizes that the consideration of the cultural significance of social inclusion and the Yazidi people’s distinctive intergenerational trauma is imperative for understanding the prevalence of depression amongst Yazidi women IDPs [41, 43]. The long history of various groups perpetrating 74 genocides against the Yazidi people over the past 800 years manifests in a myriad of forms, including collective and intergenerational trauma [42, 43].

The present study identified suicidal thoughts to be prevalent among the study population with 61.5% of Yazidi women reporting the thought of harming themselves ‘‘quite often’’ and only 2.5% reporting no thought of harming themselves. This finding is consistent with literature showing increased of suicide amongst Yazidi women [19]. In addition to historical trauma, exposure to potential traumatic events, and current living conditions in camps, the prevalence of suicidal thoughts may stem from the feelings of guilt and worthlessness, which may be exacerbated by social isolation and disruption of social support networks [19, 27, 28, 44, 45]. Shame, the fear of social ostracization, and feelings of loneliness may partly explain the high prevalence of suicidal thoughts in the Yazidi women found in the present study [46]. These findings draw attention to the importance of social support and psychosocial first aid for Yazidi IDPs during and after pregnancy [47, 48].

Additionally, this study found that perinatal depressive symptoms was statistically significantly associated with loss of a spouse. Yazidi women in Iraq, particularly in IDP camps, without a spouse may lack economic, social, and political resources, and this loss of economic security and partnership may exacerbate stress levels [46], this finding is consistent with Mohammad et al. (2018) who found that higher levels of perinatal depressive symptoms are associated with low social support and low monthly income [44], and in another study in KR-I, women with higher socioeconomic status have reported lower perinatal depressive symptoms levels [45]. A strong psychosocial support system provided by family, friends, and medical providers could act as a buffer to protect against deleterious health outcomes. The impacts of the lack of a strong social support system on the development of perinatal depressive symptoms could serve as a topic of inquiry for future studies in the field.

The present study found an association between health-related problem(s) during pregnancy with perinatal depressive symptoms. A study of postpartum depression for Syrian women in Damascus found an association between health problems during the last pregnancy and perceived exposure to more life stressor, as well as a protective effect of antenatal care on postpartum depression [49]. We also found that first time pregnancies were associated with higher risk of perinatal depressive symptoms. Yazidi IDPs may face limited access to sexual and reproductive health education, resources, and protection from gender-based violence. Future research should explore what factors may contribute to perinatal depressive symptoms during first time pregnancies among IDPs, for instance experiences with unplanned pregnancies, pregnancies resulting from rape, and pregnancies resulting from child marriage, in order to implement protections and provide targeted support efforts. Perinatal depressive symptoms was statistically significantly associated with younger age, lower level of education, and experiencing miscarriage or abortion, with older age and a higher level of education having protective effects on perinatal depressive symptoms. These findings are not represented in the broader literature and should be considered for areas of investigation for future studies.

While the EPDS has been adapted in many languages and used as a simple and cost-effective screening instrument for perinatal depressive symptoms, some reports have drawn attention to challenges in its implementation and validity in culturally and linguistically diverse populations [50]. Sociocultural factors are critically important to understanding how to adapt and tailor mental health screening and treatment interventions, particularly in humanitarian settings [51–53]. Ultimately, the decision to use the EPDS in the present study was informed by our research team’s expertise in humanitarian health and cross-cultural perinatal health research, in addition to our experience using this tool prior to the study as part of the larger mixed-methods study of Yazidi women’s experiences with pregnancy, birth, and breastfeeding in IDP camps in remote regions of the KR-I (data not presented). The scalability of the EDPS as a perinatal and postpartum mental health screening tool for IDPs in Iraq would be further enhanced by rigorous validation studies, including studies of the validity and reliability of the EPDS anxiety sub-scale and suicidality screening items for conflict affected IDP populations in Iraq, along with focused qualitative studies of Yazidi perceptions of perinatal mental health issues and experiences.

## Limitations

The present study adds an important contribution to the literature, documenting the complex mental health needs of internally displaced Yazidi women living in IDP camps throughout remote areas of KR-I. However, this study is constrained by a number of limitations. Conducting research with extremely marginalized and vulnerable populations affected by conflict and complex humanitarian crises requires attentiveness to several important ethical issues. The study was designed to be responsive to the difficult conditions of conducting research in IDP camps in the KR-I. These conditions precluded gathering more in-depth and detailed information regarding individual participants’ mental health histories, specific exposures to trauma, other measures of trauma and mental health conditions, and demographic information. For these reasons, we were not able to conduct robust statistical analyses to identify other predictors of perinatal depression risk, nor were we able to link our EPDS results to other mental health screening and diagnostic instruments. While the EPDS was an acceptable instrument to use for the present study, more comprehensive and culturally responsive perinatal mental health screening and treatment supports are needed for Yazidi IDPs.

## Conclusions

The current study demonstrates a high prevalence of perinatal depressive symptoms and suicidal ideation amongst Yazidi women IDPs in the KRI. The provision of psychosocial services that address both immediate perinatal health care and the intergenerational effects of trauma is crucial. Evaluation of such services will inform program implementation by providing evidence for what forms of trauma-informed healthcare are most effective in the context of Yazidi women during and after their first pregnancy. Additionally, future research should examine the potential of strong social support networks to serve as a buffer and act as a protective force against perinatal depressive symptoms and other mental disorders in the context of Yazidi IDPs. The present paper contributes several novel findings that require additional research that will enhance psychosocial support services provided to Yazidi IDPs.

## Data Availability

The datasets generated during the current study are not publicly available because consent to share raw data was not obtained from participants. This is due to the sensitive nature of the information collected in this study, and due to the highly vulnerable status of the study’s participants.

## Abbreviations

DSM: Diagnostic and Statistical Manual of Mental Disorders
EPDS: Edinburgh Postnatal Depression Scale
IDP: Internally displaced person
ISIS: Islamic State of Iraq and Syria
KRI: Kurdistan Region of Iraq
LMIC: Low- and middle-income countries
PMD: Perinatal mood disorder
PTE: Potential traumatic event
WHO: World Health Organization
